# Selection of Optimal Ancestry Informative Markers for Classification and Ancestry Proportion Estimation in Pigs

**DOI:** 10.3389/fgene.2019.00183

**Published:** 2019-03-11

**Authors:** Zuoxiang Liang, Lina Bu, Yidi Qin, Yebo Peng, Ruifei Yang, Yiqiang Zhao

**Affiliations:** ^1^Beijing Advanced Innovation Center for Food Nutrition and Human Health, China Agricultural University, Beijing, China; ^2^State Key Laboratory of Agrobiotechnology, College of Biological Sciences, China Agricultural University, Beijing, China

**Keywords:** ancestry informative markers, *F_ST_*, classification, pig, ancestry proportion

## Abstract

Using small sets of ancestry informative markers (AIMs) constitutes a cost-effective method to accurately estimate the ancestry proportions of individuals. This study aimed to generate a small and effective number of AIMs from ∼60 K single nucleotide polymorphism (SNP) data of porcine and estimate three ancestry proportions [East China pig (ECHP), South China pig (SCHP), and European commercial pig (EUCP)] from Asian breeds and European domestic breeds. A total of 186 samples of 10 pure breeds were divided into three groups: ECHP, SCHP, and EUCP. Using these samples and a one-vs.-rest SVM classifier, we found that using only seven AIMs could completely separate the three groups. Subsequently, we utilized supervised ADMIXTURE to calculate ancestry proportions and found that the 129 AIMs performed well on ancestry estimates when pseudo admixed individuals were used. Furthermore, another 969 samples of 61 populations were applied to evaluate the performance of the 129 AIMs. We also observed that the 129 AIMs were highly correlated with estimates using ∼60 K SNP data for three ancestry components: ECHP (Pearson correlation coefficient (*r*) = 0.94), SCHP (*r* = 0.94), and EUCP (*r* = 0.99). Our results provided an example of using a small number of pig AIMs for classifications and estimating ancestry proportions with high accuracy and in a cost-effective manner.

## Introduction

Autosomal single-nucleotide polymorphism (SNP) and insertion-deletion (InDel) are widely utilized for human ancestry inference and population assignment (Bauchet et al., 2007; Tian et al., 2009; Sun et al., 2016). Ancestry informative markers (AIMs) are genetic markers of frequency differences between populations (Shriver et al., 2003). Multiple statistics have been used to obtain AIMs, including *F* statistics (*F_ST_*), absolute allele frequency differences (*δ*), informativeness for assignment measure (*I_n_*), and principal component loading scores (Rosenberg et al., 2003; Zhang et al., 2009; Ding et al., 2011; vonHoldt et al., 2016; Barbosa et al., 2017; Peterson et al., 2017). Instead of using whole genome markers, AIMs were considered to be sufficiently accurate for ancestry inference for limited population size. Consequently, this constitutes an economical way to screen and analyze thousands of samples. Santos et al. (2016) reported that 192 AIMs selected from ∼370 K SNP data can be used to accurately estimate the ancestry proportions of three major populations in Brazil. Li et al. (2016) developed a panel of 74 AIMs to infer the ancestry proportions of 500 test individuals from 11 populations. Due to the high resolution of AIMs, a 23-AIMs panel generated by Zeng et al. (2016). distinguished four major American populations, and correctly assigned ancestry for nine additional populations (Zeng et al., 2016).

For animal population genetics, AIMs have been successfully applied to identify breeds of different varieties and to evaluate genetic compositions in hybrid populations (Dimauro et al., 2015; Bouchemousse et al., 2016). Bertolini et al. (2017) found that 96 AIMs performed well in discriminating six dairy cattle breeds. In another study, 63 AIMs selected from 427 canids were utilized to assess genetic admixture in coyotes (Monzon et al., 2014). Recently, 74 AIMs were used to calculate ancestry proportions in crossbred sheep (Awassi with two native breeds in Ethiopia), and it was found that different admixture levels of Awassi significantly affected the traits of lamb growth and ewe reproduction (Getachew et al., 2017).

The pigs (*Sus scrofa*) diverged into European and Asian wild boars during mid-Pleistocene (1.2–0.8 million years ago) (Larson et al., 2005; Frantz et al., 2013). Pig domestication in China occurred ∼9,000 years ago (Larson et al., 2005). It has been documented that Chinese domestic pigs were divided into six types according to the region of dwelling and phenotype characteristics (I–North China, II–Lower Changjiang Basin, III–Central China, IV–South China, V–Southwest, and VI–Plateau) (Li et al., 2004; Fang et al., 2005). In a recent study, Yang et al. (2017). tracked the ancestries of various Chinese breeds and identified two major distinct ancestries, which are East China (e.g., Meishan and JinHua) and South China (e.g., Luchuan and Bamaxiang) origin. In addition, genomic introgression from European commercial breeds to Chinese indigenous pigs has also been reported (Ai et al., 2013; Bosse et al., 2014; Zhu et al., 2017), making the genetic compositions of modern Chinese pigs even more complicated.

Although it has been widely applied in other animals, and it is of great importance in specific application scenarios, including market surveillance and genetic resource protection, no study currently exists that specifically addresses the problem of efficiently using AIMs for distinguishing pig breeds or for estimating ancestry proportions. Here, using ∼60 K pig SNP chip data, we searched for the optimal number of AIMs for distinguishing pigs of East China, South China, or European origin. Based on 129 selected AIMs, we estimated ancestry proportions of the above origins for other Chinese pigs. We suggested that AIMs selected from unadmixed reference populations could be used to accurately estimate ancestry proportions in hybrid populations. Our results provide a useful example of utilizing AIMs for breed classification and ancestry estimation in pigs.

## Materials and Methods

### Data Collection and Quality Control

Genotyping data of 2,113 samples were retrieved from the Dryad Digital Repository^1^. Only samples from Asian breeds, and European breeds were used in this study (a total of 1,157 samples from 71 populations, details in Supplementary Table S1). Samples and SNPs were excluded if the following criteria were met: (1) an individual contained more than 10% missing genotypes; (2) SNPs with a call rate lower than 95%; (3) SNPs with a minor allele frequency less than 0.05; (4) SNPs that were located on sex chromosomes; and (5) SNPs were not biallelic. The missing genotypes were subsequently imputed by using BEAGLE (version 3.3.2) (Browning and Browning, 2007). Finally, 45,562 SNPs and 1,155 samples remained. The 1,155 samples were then split into two datasets. For the reference set, 186 samples were chosen from 10 representative populations of the three major ancestry groups: East China pig (ECHP), South China pig (SCHP), and European commercial pig (EUCP). The 10 populations were selected based on the fact that there was no obvious admixture between populations belonging to the ECHP or SCHP group, according to a report from Yang et al. (2017). This data set is summarized in Table 1. The test dataset contained the remaining 969 samples from 61 populations (details in Supplementary Table S2). Considering the convenience of practical application, the genotype data of the test dataset were directly extracted from the raw data without phasing or imputation.

**Table 1:** Pig breeds information in the reference set.

Group	Subpopulation	Abbreviation	Number
South China pig (SCHP)	China_Bamaxiang	CNBX	16
	China_Congjiangxiang	CNCJ	16
	China_Guangdongdahuabai	CNDH	16
	China_Luchuan	CNLU	18
East China pig (ECHP)	China_Erhualian	CNEH	20
	China_Jinhua	CNJH	20
	China_Meishan	CNMS	20
European Commercial pig (EUCP)	Duroc	DUR2	20
	Pietrain	PIT1	20
	Landrace	LDR1	20


### Population Structure

Principal component analysis (PCA) was performed on ∼60 K chip data using SMARTPCA (version 6.1.4) in the reference set (Patterson et al., 2006). To confirm the unadmixed status, the unsupervised ADMIXTURE (version 1.23) (Alexander et al., 2009) was utilized to compute the ancestry proportions of samples from the reference set with the number of ancestry (K) set from K = 3 through K = 15. The ChromoPainter v2 (Lawson et al., 2012) linked model was also chosen to explore similarity/dissimilarity for individuals in the reference set. In detail, the recombination map file was generated using the script makeuniformrecfile.pl provided by fineSTRUCTURE (version 2.1.1) (Lawson et al., 2012). By utilizing a hidden Markov model profile, ChromoPainter v2 infers haplotypes of “donor” and “recipient” to create a co-ancestry matrix. Initially, 20 expectation-maximization steps were used to estimate the mutation and switch rate on 1/5 random sampling members from all individuals with all autosomes considered. The inferred mutation and switch rates for each chromosome were then averaged. Subsequently, with estimated mutation, switch rate and other default values, ChromoPainter v2 was again used to generate the co-ancestry matrix for all individuals. Finally, the MCMC algorithm implemented in fineSTRUCTURE was employed to hierarchically cluster individuals with a burn-in and runtime of 1,000,000 and 6,000,000 iterations, respectively.

### Selection of AIMs

All 186 samples in the reference dataset were used to compute *F_ST_* and *I_n_*. Candidate SNPs were selected from the AIMs algorithm selector that was implemented in AIMs_generator.py from ANTseq pipeline^2^. Specifically, we firstly excluded SNPs in high-linkage disequilibrium (LD) by selecting only one SNP in a strong LD (*r*^2^ > 0.3) region and within 500 kb distance. Within each group, SNPs that exhibited heterogeneous frequencies among populations were further excluded based on a Chi-squared test (Galanter et al., 2012). Secondly, *F_ST_* and *I_n_* were computed for each of the three paired groups : ECHP vs. EUCP, SCHP vs. EUCP, and ECHP vs. SCHP (Rosenberg et al., 2003).

### Group Classification With Minimum AIMs

Using the reference dataset, we first compared the discriminatory power of the AIMs selected by *F_ST_* or *I_n_*. Binary classification for the three paired groups were performed separately. For each paired group, we started by selecting the top two through top 30 AIMs, with an increment of one AIM. Samples in the corresponding paired group were randomly split into two proportions: 75% for training, 25% for testing, and this operation was repeated 50 times. *GridSearchCV* implemented in the Scikit-learn (version 0.18) package was then used to determine the optimal parameters for a support vector machine (SVM) classifier (Da Mota et al., 2014). The parameters for SVM are summarized in Supplementary Table S3. For the model with optimal parameters, the accuracy of classification was evaluated by the mean of the Matthews correlation coefficient (MMCC) for 50 repeats as follows:

$$\text{MMCC}=\left[\sum_{\text{i}=1}^{50}\frac{{\text{TP}}_{\text{i}}\times {\text{TN}}_{\text{i}}{\text{-FP}}_{\text{i}}\times {\text{FN}}_{\text{i}}}{\sqrt{\left({\text{TN}}_{\text{i}}{\text{+FN}}_{\text{i}}\right)\left({\text{TP}}_{\text{i}}{\text{+FP}}_{\text{i}}\right)\left({\text{TP}}_{\text{i}}{\text{+FN}}_{\text{i}}\right)\left({\text{TN}}_{\text{i}}{\text{+FP}}_{\text{i}}\right)}}\right]\times \frac{1}{50}$$

where TN_i_ and FN_i_ are the number of true negatives and false negatives, and TP_i_ and FP_i_ are the number of true positives and false positives, for each run.

To determine the minimum number of AIMs for distinguishing ECHP, SCHP, and EUCP simultaneously, a multiclass approach of one-vs.-rest SVM was employed on reference dataset (Hong and Cho, 2008). Similarly, we began by selecting the top two through top 200 AIMs from each of the paired groups, with an increment of one AIM, resulting in 199 AIM sets in total. In each set, AIMs selected from the three paired groups were merged and duplicated AIMs were removed (Supplementary Table S4). Since MMCC was not designed for evaluating the accuracy of multiclass classification, confusion matrix, Cohen’s kappa statistic and balanced error rate were used instead to evaluate the classification accuracy. Higher Cohen’s kappa but lower balanced error rate indicated higher accurate classification. We again utilized *GridSearchCV* to estimate the best parameters for one-vs.-rest SVM, the parameters of which are summarized in Supplementary Table S3. We also generated random SNP sets of equal number from the whole genome for comparison of discriminatory power to the selected AIMs.

### Ancestry Inference With Optimal AIMs

AIMs have been widely used to estimate ancestry proportions in hybrid populations, even in cases in which they were selected from unadmixed populations. Based on selected AIMs, to estimate ancestry proportions of possible admixed pig populations, we employed a strategy that was similar to that used in a previous study by Pardo-Seco et al. (2014). We first generated pseudo admixed individuals by randomly selecting genotypes of selected AIMs from samples in the reference data set with equal proportions. Therefore, the expected ancestry proportions of these pseudo admixed individuals were 1/3 (∼0.3333) from each group (ECHP, SCHP, and EUCP). For each of the 199 AIM sets generated from the above, 1,000 simulations were performed. Supervised ADMIXTURE (K = 3) was used to estimate the ancestry proportions. The performances were evaluated by the mean and the coefficient of variation (CV) of the estimated ancestry proportions. The CV of estimated ancestry proportions against the number of AIMs was fitted by the Curve Expert 1.4 program^3^. The optimal number of AIMs was determined by selecting the slope of the tangent threshold of the curve of which stable performance was observed beyond that point. To add an additional validation, we simulated pseudo admixed individuals with random ancestry proportions using the determined optimal number of AIMs. The ancestry proportions of ECHP, SCHP, and EUCP were randomly assigned with a minimum proportion set to 10%.

On the basis of the AIMs selected in the last step, we performed ancestry inference for the 969 individuals in the test dataset by supervised ADMIXTURE. The performance was evaluated by Pearson correlation coefficient between the genome-wide SNPs and the optimal number of AIMs.

## Results

### Population Structure of Reference Populations

**FIGURE 1:**
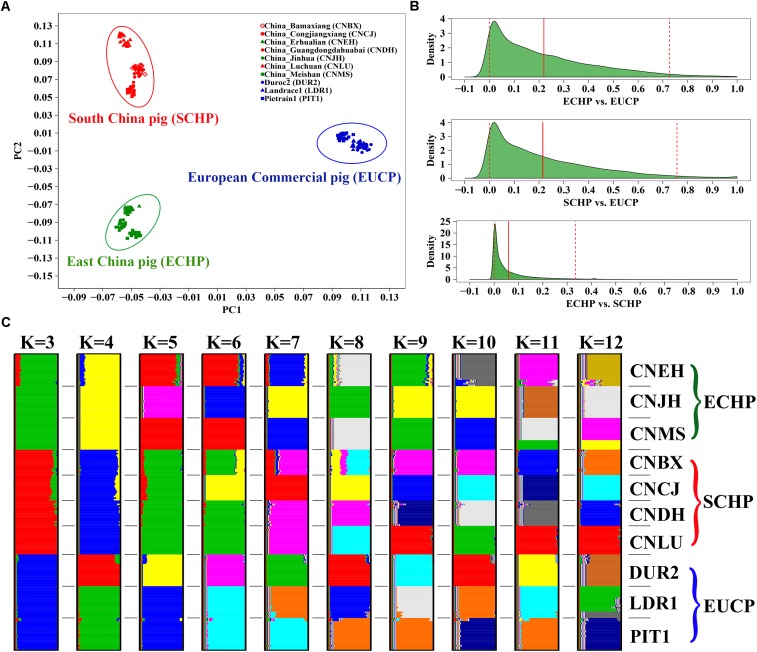
Population structure of 10 breeds in the reference data set. **(A)** Principal component analysis (PCA) of ∼60 K chip data. **(B)** The genome-wide *F_ST_* distribution for the three paired groups: ECHP vs. EUCP, SCHP vs. EUCP and ECHP vs. SCHP. The red vertical line represents the mean of *F_ST_* distribution. The dashed vertical lines represent 2.5 and 97.5% percentile of *F_ST_* distribution. **(C)** ADMIXTURE clustering of ∼60 K chip data when K = 3–12. CNBX, China_Bamaxiang; CNCJ, China_Congjiangxiang; CNLU, China_Luchuan; CNDH, China_Guangdongdahuabai; CNJH, China_Jinhua; CNEH, China_Erhualian; CNMS, China_Meishan; DUR2, Duroc2; PIT1, Pietrain1; LDR1, Landrace1. Color codes for large braces are as follows, green: East China pig (ECHP); red: South China pig (SCHP); blue: European commercial pig (EUCP).

Populations in the reference set were supposed to be least admixed. We did observe that ECHP, SCHP, and EUCP were well separated in a principal component plot (Figure 1A). The genome-wide *F_ST_* distribution (Figure 1B) showed higher differentiation both between ECHP vs. EUCP (mean = 0.2197, 95% CI 0.0006–0.7267) and SCHP vs. EUCP (mean = 0.2153, 95% CI 0.0005–0.7570), while the differentiation between ECHP vs. SCHP (mean = 0.0588, 95% CI 0–0.3342) was noticeably less pronounced. By using ADMIXTURE, all breeds were well divided into anticipated groups (Figure 1C) when K = 3, in accordance with the previous study by Yang et al. (2017). When K = 10, 10 populations could be separated clearly, consistent with our expectation that the 10 populations were least admixed (Supplementary Figure S1).

**FIGURE 2:**
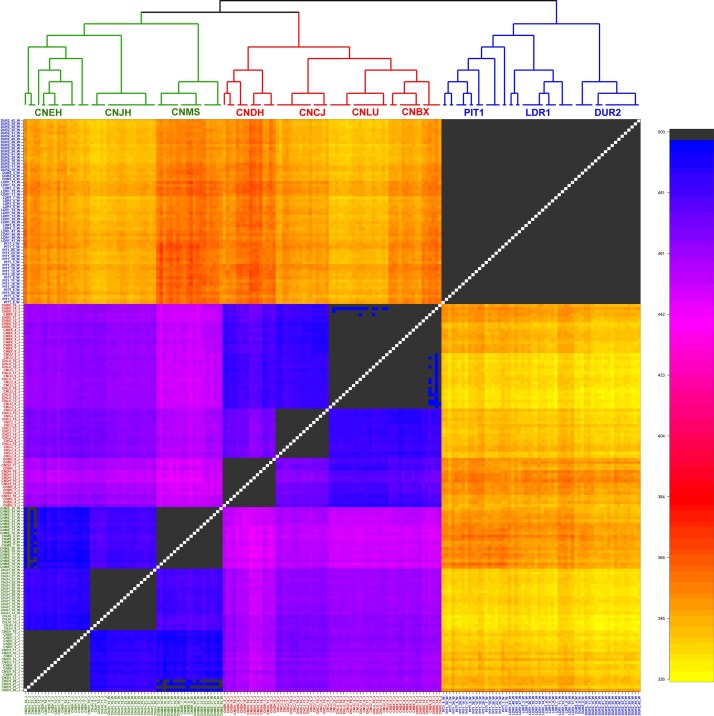
fineSTRUCTURE analysis in the reference dataset. The heatmap shows the number of shared genetic chunks copied from a donor genome (column) to a recipient genome (row). CNBX, China_Bamaxiang; CNCJ, China_Congjiangxiang; CNLU, China_Luchuan; CNDH, China_Guangdongdahuabai; CNJH, China_Jinhua; CNEH, China_Erhualian; CNMS, China_Meishan; DUR2, Duroc2; PIT1, Pietrain1; LDR1, Landrace1. Color codes are as follows, green: East China pig (ECHP); red: South China pig (SCHP); blue: European commercial pig (EUCP).

For further quantification, the ChromoPainter v2 and fineSTRUCTURE programs were employed to check the relationship among these breeds considering LD. As shown in the coancestry heatmap (Figure 2), individuals within each group exhibited a homogeneous pattern, and those from the same group shared more genetic chunks than from other groups. In particular, the EUCP had a negligible degree of coancestry with individuals from Chinese indigenous breeds. The sample from ECHP and SCHP showed a higher degree of coancestry, but individuals from the same group still tended to cluster together more than between groups. In summary, the results suggested that the samples in the reference dataset exhibited a negligible level of admixture.

### Group Classification Using AIMs

In order to build an effective set of AIMs, we firstly compared the performance of *F_ST_* statistics and *I_n_* statistics. For a paired group of ECHP vs. EUCP and SCHP vs. EUCP, a minimum of two AIMs were found to be sufficient to result in a perfect separation (MMCC = 1), either by selecting the top *F_ST_* or by top *I_n_* statistics (Supplementary Figure S2). However, to separate ECHP vs. SCHP, at least four AIMs were required by using *F_ST_*, or at least five were required by using *I_n_*. For AIMs selected by *F_ST_* or *I_n_*, we found that informative AIMs selected by *I_n_* were largely overlapped with AIMs selected by *F_ST_*, indicating that *F_ST_* is at least as informative as *I_n_*. Therefore, the following analyses were based only on AIMs selected by *F_ST_*.

**FIGURE 3:**
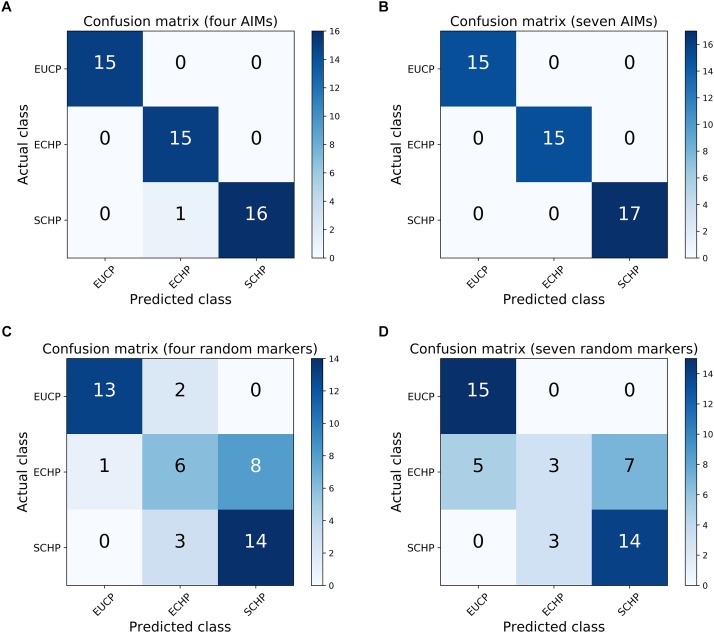
Confusion matrices for the one-vs.-rest SVM classifier. **(A)** The performance of four AIMs. **(B)** The performance of seven AIMs. **(C)** The performance of four random markers that is sampled from whole genome data. **(D)** The performance of seven random markers.

Next, we attempted to identify the number of AIMs which could be used to separate ECHP, SCHP and EUCP simultaneously using a multiclass approach. As described in Materials and Methods, top ranked two to 200 AIMs were sequentially selected from ECHP vs. EUCP, SCHP vs. EUCP and ECHP vs. SCHP, respectively, resulting in 199 AIM sets of increasing number (Supplementary Table S4). AIMs in each set were merged and deduplicated. For example, for the largest set, 171 out of 200 AIMs were shared between ECHP vs. EUCP and SCHP vs. EUCP (Supplementary Figure S3), 12 out of 200 AIMs were the shared between SCHP vs. EUCP and ECHP vs. SCHP, and 14 out of 200 AIMs were shared between ECHP vs. EUCP and ECHP vs. SCHP. All 199 AIM sets were fed to a one-vs.-rest SVM classifier. As show in Figure 3 and Supplementary Table S5, seven AIMs were sufficient to completely separate ECHP, SCHP and EUCP with the Cohen’s kappa = 1 and balanced error rate = 0. The detailed information of seven AIMs were summarized in Table 2 and Supplementary Table S7.

**Table 2:** The pairwise *F_ST_* values for the 129 AIMs.

SNP	Chr	Position	ECHP vs. EUCP	SCHP vs. EUCP	ECHP vs. SCHP
ALGA0003690	1	64094344	0.4724	0.0300	0.3069
MARC0023378	1	148309548	0.9672	1	0.0084
INRA0004282	1	149824800	0.9672	1	0.0084
DRGA0001542	1	150801717	0.9672	0.9556	0.0005
INRA0004312	1	152175324	0.9672	1	0.0084
H3GA0002811	1	153144281	0.9508	0.9835	0.0084
INRA0004460	1	158429254	0.9028	0.9355	0.0084
ASGA0004738	1	159450284	0.9028	0.9355	0.0084
MARC0036323	1	162068596	0.9028	0.9355	0.0084
H3GA0002947	1	162610557	0.9508	0.9835	0.0084
M1GA0001158	1	163303972	0.9672	1	0.0084
**ASGA0005014**	**1**	**179090814**	**0.0534**	**0.9069**	**0.6833**
DRGA0001670	1	192768198	0.7230	0.9835	0.0811
DRGA0001766	1	201005516	0.0390	0.5438	0.4165
INRA0005593	1	211778061	0.9355	1	0.0169
INRA0005652	1	214740742	0.9347	0.9512	0.0042
ALGA0007467	1	215925031	0.9347	0.9512	0.0042
ALGA0007539	1	220517767	0.1429	0.0826	0.3608
M1GA0002066	1	307474784	0.3636	0.0154	0.3125
H3GA0005443	1	311685793	0.9185	0.9512	0.0084
ASGA0102470	2	2261977	0.9355	0.9355	0
**ASGA0008848**	**2**	**7823419**	**0.0031**	**0.5319**	**0.5905**
ASGA0091359	2	96158022	0.0084	0.3750	0.3460
ASGA0012212	2	140996142	0.0018	0.4149	0.3737
ALGA0016543	2	145257971	0.2061	0.0234	0.3266
MARC0065978	3	55717402	1	0.9412	0.0154
ALGA0019771	3	77636015	0	0.4293	0.4293
DIAS0003766	3	81986256	0.9512	0.9512	0
ALGA0107390	3	86085644	0.9355	0.9355	0
ASGA0101711	3	123638149	0.9344	0.9373	0
ASGA0016597	3	132275049	0.0573	0.6011	0.3479
ALGA0024245	4	30874523	0.0003	0.3275	0.3426
**ASGA0019402**	**4**	**41300090**	**1**	**0.9702**	**0.0076**
DRGA0004757	4	43615316	0.9512	0.9512	0
ALGA0025201	4	61401615	0.0061	0.4776	0.3969
INRA0014351	4	66793036	0.9512	1	0.0127
MARC0090092	4	68409038	0.9512	1	0.0127
**INRA0014612**	**4**	**73495852**	**1**	**1**	**0**
ASGA0021073	4	103804488	0.0390	0.4081	0.5349
ALGA0031043	5	19526692	0.1747	0.0480	0.3044
ALGA0031742	5	39327879	0.9512	0.9512	0
DRGA0005727	5	40915894	0.9512	0.4550	0.2000
INRA0019276	5	42724575	0.9671	0.9671	0
ASGA0025483	5	44622629	0.9671	0.9671	0
DRGA0005762	5	45252255	0.9671	0.9671	0
**DRGA0005767**	**5**	**46381993**	**1**	**1**	**0**
ALGA0031838	5	47243385	1	1	0
**MARC0046863**	**5**	**48236817**	**1**	**1**	**0**
DRGA0005792	5	49040715	1	1	0
ALGA0031894	5	50991368	0.9671	0.9671	0
INRA0019346	5	52798149	1	1	0
ALGA0032094	5	62157042	0.0260	0.4682	0.3097
ALGA0108031	5	67710315	0.1429	0.0491	0.3005
ALGA0032500	5	68352730	0.4423	0.0205	0.3048
ASGA0026083	5	69584032	0.3413	0	0.3461
INRA0020365	5	96352161	1	0.9702	0.0076
ALGA0037079	6	133726563	0.9355	0.9355	0
ALGA0117693	6	148742719	0.9185	0.9512	0.0084
**ASGA0094022**	**6**	**151217323**	**0.0784**	**0.3521**	**0.6331**
MARC0041948	6	152894649	0.3793	0	0.3793
MARC0115216	7	3195217	0.9348	0.9835	0.0127
DBKK0000285	7	60252514	0.4906	0	0.4906
H3GA0021983	7	67646165	0.3886	0.0010	0.3767
ALGA0042537	7	77009237	0.0114	0.4408	0.3630
DRGA0007820	7	77973037	0.0402	0.5930	0.4218
DIAS0000146	7	109783926	0.9512	0.9068	0.0115
ALGA0045522	7	127332840	0.6741	0.0977	0.3392
H3GA0024530	8	23601551	0.1984	0.0820	0.4514
BGIS0004952	8	39300733	0.9512	0.9512	0
ASGA0038742	8	41242759	0.9835	0.9835	0
ALGA0047876	8	52213568	0.0084	0.4505	0.4206
H3GA0024898	8	61863927	1	0.5715	0.1579
ALGA0047992	8	65489064	0.0169	0.5903	0.5294
INRA0029873	8	69998730	0.0014	0.3115	0.3256
ALGA0048179	8	77021275	0.9835	0.9247	0.0154
ALGA0048253	8	78671695	1	0.9850	0.0038
ASGA0039683	8	121829327	0.9671	0.9671	0
ASGA0039832	8	130909240	0.9670	0.9835	0.0042
INRA0030531	8	131517760	0.9835	1	0.0042
H3GA0025494	8	135031919	0.0057	0.2447	0.3073
ASGA0095368	8	145709748	0.0455	0.2561	0.4043
H3GA0055769	9	12292138	0.1918	0.0313	0.3512
ALGA0119045	9	15055604	0.0292	0.4774	0.3070
BGIS0007566	9	53579054	0.9672	0.9850	0.0017
ASGA0043529	9	66808115	0.0057	0.2435	0.3044
ASGA0096819	9	73486663	0.0042	0.5017	0.5172
ASGA0043850	9	85054037	0.1853	0.0811	0.4384
ALGA0054899	9	129924803	0.9190	0.9685	0.0083
H3GA0028160	9	130830299	0.9355	0.9805	0.0083
H3GA0053792	10	13926830	0.0014	0.3924	0.3550
ALGA0057773	10	25842467	0.2107	0.0296	0.3532
M1GA0014504	11	55295	1	0.9556	0.0115
INRA0036515	11	55728615	0.7857	0.1899	0.3166
M1GA0016423	12	23116412	0.9671	0.9671	0
MARC0072483	12	46436962	0.2169	0.1199	0.5465
ASGA0104770	12	60372315	0.0883	0.1469	0.3674
MARC0010739	13	40671308	0.9512	0.5537	0.1429
ALGA0069709	13	42825042	0.9512	0.9068	0.0115
MARC0094198	13	43808238	1	0.9556	0.0115
ALGA0114810	13	49676914	0.4135	0.0134	0.3055
ASGA0057953	13	71998014	0.0526	0.1905	0.3333
ALGA0070726	13	73168055	0.5376	0.0017	0.5543
INRA0040831	13	115237462	0.9348	0.9835	0.0127
INRA0040844	13	117860412	0.9348	0.9835	0.0127
INRA0040883	13	124139277	0.9185	0.9512	0.0084
ALGA0076648	14	30849871	0.0547	0.5746	0.3880
DBMA0000255	14	131114363	0.5385	0.0512	0.3135
M1GA0019170	14	135148446	0.9512	0.9512	0
ALGA0113804	15	2724715	0.1694	0.0563	0.3810
INRA0048834	15	14037549	0.3636	0.0031	0.3135
ALGA0084945	15	40545702	0.0069	0.4234	0.3399
ALGA0088237	15	148849949	0.8321	0.0550	0.5715
MARC0040430	16	2142400	0.2813	0.0230	0.4293
ASGA0071886	16	2745009	0.1903	0.0568	0.3759
ASGA0072342	16	14465203	0.1594	0.0716	0.3679
H3GA0046303	16	24940106	0.0899	0.8021	0.4683
ALGA0090172	16	35245008	0.0344	0.1554	0.3116
MARC0080217	16	58476421	0.9835	0.9702	0.0010
ALGA0094674	17	39199731	0.0243	0.2865	0.4208
ALGA0095308	17	50025811	0.9355	0.9355	0
MARC0041179	18	617438	0.2592	0.8749	0.3044
ALGA0097196	18	16559678	1	1	0
ASGA0079061	18	17817003	0.6083	0.0472	0.3757
ASGA0079737	18	44927369	0.9671	0.9671	0
DBWU0000187	18	46589140	0.0006	0.2977	0.3202
M1GA0023257	18	48941351	0.9512	0.9068	0.0115
ALGA0098723	18	54367433	0.9348	0.9835	0.0127
ALGA0098742	18	55295182	0.9672	1	0.0084
ASGA0080420	18	58822946	0.9355	0.9556	0.0010


*The information of the seven AIMs which could completely separate ECHP, SCHP and EUCP are indicated in bold font. Chr, chromosome.*

### Accurate Ancestry Proportion Estimation Using AIMs

AIMs selected from unadmixed populations were reported to be successfully applied to estimate ancestry proportions in admixed populations (Lee et al., 2012; Maples et al., 2013). To validate practicability in our study, we performed data simulation. If the study is practical, we should observe high consistency between simulated and estimated ancestry proportions. For each AIM set, the supervised ADMIXTURE was used to calculate ancestry proportions in 1,000 simulations. For each simulation, genotype of 60 samples selected from ECHP, SCHP and EUCP were randomly mixed for each AIM.

**FIGURE 4:**
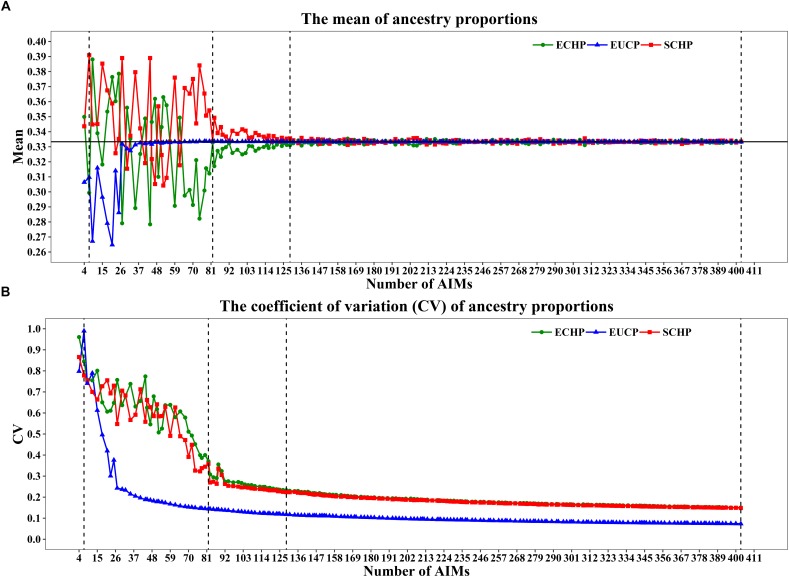
Ancestry inference on simulated individuals from the 199 AIM sets. In each set, 1,000 simulations were performed by a python script and the ancestries were inferred by supervised ADMIXTURE. Vertical dashed lines represent four AIM sets: seven AIMs,82 AIMs, 129 AIMs and 403 AIMs. **(A)** The mean of ancestry proportions for the three groups: ECHP (green), SCHP (red) and EUCP (blue). The black horizontal line represents the expected value (∼0.3333) of each ancestry. **(B)** The coefficient of variation (CV) of ancestry proportions for the three groups.

As shown in Figure 4A,B, when 80 or fewer AIMs were included, large differences between the mean of estimated and expected value (∼0.3333) were observed. For example, the seven AIMs worked perfectly for classification were not sufficient to infer the ancestry proportions accurately: ECHP (mean = 0.2994, coefficient of variation (CV) = 0.8450), SCHP (mean = 0.3909, CV = 0.7783) and EUCP (mean = 0.3097, CV = 0.9895). However, by including top 82 AIMs or more, the estimated proportions gradually converged to the expected values (Figure 4A). Same tendency for the CV plot in which the CV decreased as the number of AIMs increased (Figure 4B).

**FIGURE 5:**
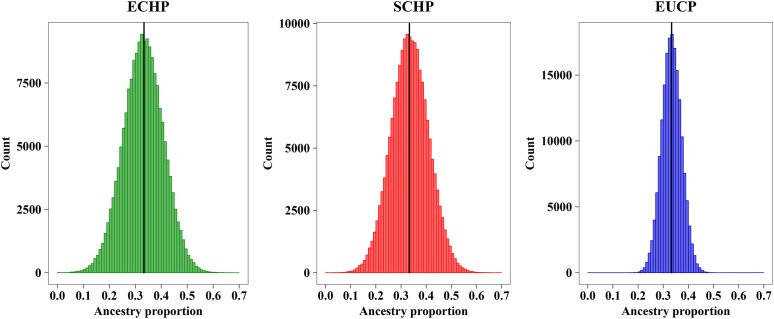
Ancestry inference on simulated individuals from 129 AIMs. The black horizontal line represents the expected value (∼0.3333) of each ancestry. Color codes are as follows, green: ECHP; red: SCHP; blue: EUCP.

In order to determine the optimal AIM set, we fitted the CV curves in Figure 4B with a reciprocal logarithmic function (Supplementary Figure S4) for AIMs between 82 and 403. Since the tangent to the curve gets infinitely close to zero, we determined an arbitrary threshold of –0.0004, which corresponds to the set of 129 AIMs, by considering both the stability of the CV value and the genotyping cost for SNPs (Supplementary Table S6). The AIM set of 129 performed well in ancestry inference for simulated samples (Figure 5), which resulted in ECHP: mean = 0.3310, standard deviation (std) = 0.0772; SCHP: mean = 0.3356, std = 0.0751; and EUCP: mean = 0.3334, std = 0.0394. We also observed that the performance of 129 AIMs set showed very limited difference to that of 403 AIMs set, suggesting the 129 AIMs set was optimal (Supplementary Table S6).

Considering the practicability of the 129 AIMs set, we next simulated pseudo admixed individuals with unequal random ancestry proportions using the same AIMs. we first produced 10 random ancestry proportions for each three groups, and then ran 1,000 simulations on each three ancestry proportions. For each simulation, 60 pseudo admixed individuals were generated. As shown in Table 3, the 129 AIMs worked very well, even for samples of random ancestry proportions.

**Table 3:** Simulation of random ancestry proportions using the 129 AIMs.

	ECHP			SCHP			EUCP		
	expectation	mean	95% CI	expectation	mean	95% CI	expectation	mean	95% CI
1	0.2083	0.2015	0.0495–0.3528	0.4333	0.4393	0.2914–0.5864	0.3583	0.3585	0.2818–0.4366
2	0.1583	0.1504	0.0000–0.2954	0.3000	0.3076	0.1652–0.4486	0.5417	0.5420	0.4617–0.6210
3	0.2500	0.2440	0.0968–0.3925	0.6000	0.6056	0.4604–0.7486	0.1500	0.1501	0.0924–0.2119
4	0.7333	0.7344	0.6049–0.8512	0.1167	0.1151	0.0000–0.2365	0.1500	0.1501	0.0920–0.2116
5	0.5083	0.5121	0.3612–0.6553	0.3750	0.3719	0.2283–0.5173	0.1167	0.1167	0.0643–0.1735
6	0.3500	0.3521	0.2009–0.5014	0.4667	0.4640	0.3153–0.6123	0.1833	0.1834	0.1210–0.2489
7	0.4000	0.4027	0.2509–0.5520	0.4500	0.4466	0.2987–0.5942	0.1500	0.1500	0.0912–0.2135
8	0.2250	0.2184	0.0692–0.3681	0.5333	0.5394	0.3932–0.6839	0.2417	0.2422	0.1729–0.3141
9	0.7083	0.7100	0.5830–0.8284	0.1167	0.1156	0.0000–0.2391	0.1750	0.1749	0.1132–0.2398
10	0.2333	0.2272	0.0818–0.3765	0.6083	0.6145	0.4699–0.7566	0.1583	0.1583	0.0998–0.2228


As anticipated, using the 129 AIMs (Table 2 and Supplementary Table S7), PCA demonstrated that 10 populations were clearly divided into three corresponding groups (Supplementary Figure S5). Interestingly, in comparison to Figure 1A, substructure within populations at each group was less obvious.

### Ancestry Proportion Estimation for the Test Dataset

It has been reported that some Asian pig breeds were admixed with European domestic breeds, and especially with commercial breeds. For instance, eight Asian breeds (Korean local breed (KPKO), Thailand local breed (THCD), China Lichahei (CNLC), China Sutai (CNST), China Kele (CNKL), China Guanling (CNGU), China Leanhua (CNLA), and China Minzhu (CNMZ)) have been reported to be introgressed by at least 20% from European ancestry (Yang et al., 2017). In order to symmetrically identify and quantify the introgression, we utilized the 129 selected AIMs to estimate the ancestry compositions of another 969 samples from 61 populations that are possibly admixed at least to a certain extent.

**FIGURE 6:**
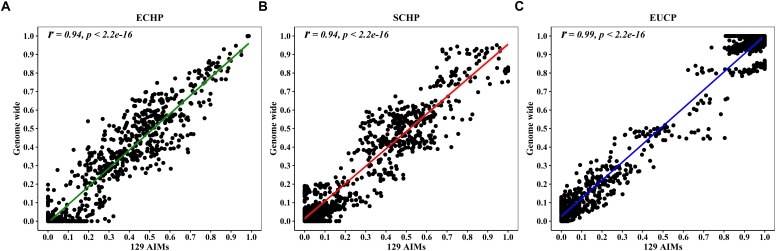
Pearson correlation between ancestries estimated by 129 AIMs and ∼60 K chip data. **(A)** Correlation for ECHP ancestry. **(B)** Correlation for SCHP ancestry. **(C)** Correlation for EUCP ancestry.

**FIGURE 7:**
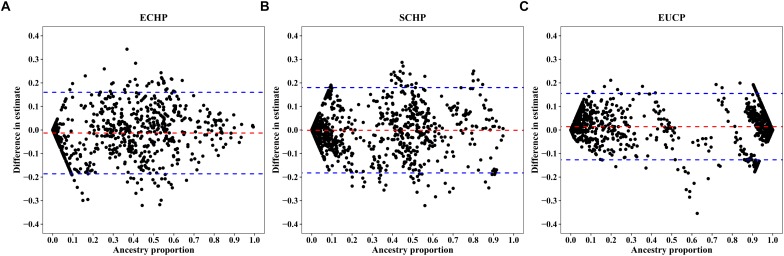
Bland-Altman plots showing difference between individual ancestry inference. The *x*-axis represents **(A)** ECHP, **(B)** SCHP, and **(C)** EUCP ancestry proportion estimated by genome-wide, respectively. The *y*-axis represents the difference in estimates between genome-wide and 129 AIMs data. The red and blue dashed lines are mean and 95% confidence intervals, separately.

**FIGURE 8:**
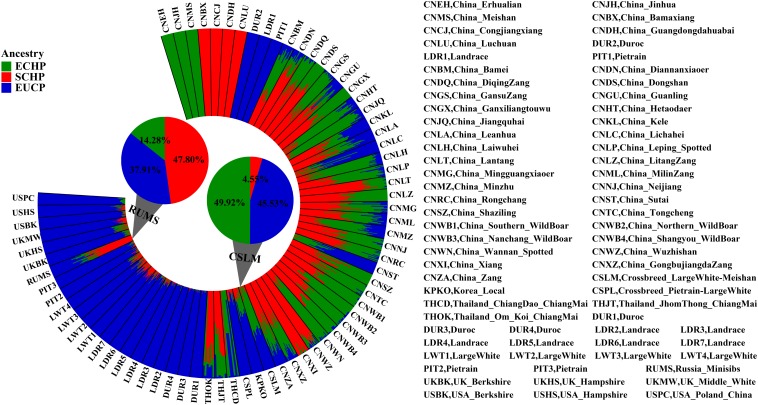
Ancestry proportions estimated by supervised ADMIXTURE at K = 3. The height of each bar represents three ancestry proportions [ECHP (green), SCHP (red) and EUCP (blue)] in one population. The mean proportion of each ancestry in RUMS and CSLM is highlighted with pie charts, respectively.

Overall, by using the supervised ADMIXTURE, we found a strong correlation (Figure 6) between ancestry proportions calculated by 129 AIMs and those calculated by all ∼60 K chip data at the individual level. Bland–Altman plot also showed agreements on ancestry proportion estimated between genome-wide and 129 AIMs data (Figure 7). For breeds that were known to be introgressed from EUCP, we obtained reasonable results. As shown in Figure 8 and Supplementary Table S8, the estimation of the mean of three ancestry proportions in the CNMZ population by using 129 AIMs (ECHP:0.5325, SCHP:0.2456, EUCP:0.2219) was similar to the estimation of the mean of three ancestry proportions in the CNMZ population by using ∼60 K SNP data (ECHP:0.6457, SCHP:0.1291, EUCP:0.2252). The LargeWhite-Meishan crossbreed (CSLM), which has been documented as an F1 generation from LargeWhite × MeiShan, our ancestry proportion estimation from the 129 AIMs (ECHP:0.4992, SCHP:0.0455, EUCP:0.4553) was consistent with the expectation, and similar to the result from ∼60 K SNP data (ECHP:0.5128, SCHP:0.0020, EUCP:0.4852). In another case, Russia Minisibs (RUMS), which has been reported to possess approximately half European ancestry, we also obtained a high level of EUCP ancestry using either 129 AIMs (ECHP:0.1428, SCHP:0.4780, EUCP:0.3791) or ∼60 K SNP data (ECHP:0, SCHP:0.5349, EUCP:0.4651).

## Discussion

Since the 19th century, pig breeders in the West have used Chinese pigs to hybridize with European pigs to improve their breeding stock (Groenen, 2016). Bianco et al. (2015) found that European domestic pigs have 20% genomic introgression from Asian pigs. On the other hand, Yang et al. (2017) reported that European pigs contributed at least 20% to eight Asian breeds. In recent years, evidence has been presented that local Chinese farmers cross local pigs with imported commercial pigs (Berthouly-Salazar et al., 2012). Introgression introduces new genetic materials, which might help to improve certain characteristics, especially production performance. Unfortunately, introgression, in either a narrow sense, as an admixture with foreign breeds, or in a broad sense, as an admixture with breeds from different areas within a nation, also introduces “genetic pollution” which is hardly avoidable. For example, in recent study, Zhang et al. found that almost all Chinese indigenous chickens have gene introgression from commercial broiler (Zhang et al., 2019).

Since the indigenous pork are sold at higher price than that of European commercial pigs in China, false propaganda, shoddy phenomenon on the market began to rise. Significant attention has been paid to the issue of pork adulteration, however, at this stage, the work of identification was mostly based on intuitions and experiences from the customer side (Dai et al., 2009; Kwon et al., 2017). Fortunately, pig products from the 10 breeds in our reference set are dominant in China (Bosse et al., 2015; Gong et al., 2018; Zhao et al., 2018), our method thus constitutes a promisingly effective way in detection of pork adulteration at DNA level in market surveillance. From the view of a researcher, in genome-wide association studies, different genetic ancestries between case and control will lead to population stratification. Therefore, if selecting the samples of similar ancestry proportions or considering ancestry as covariates in the regression model to adjust stratification, it would help to reduce false positives(Qin et al., 2014).

Overall, it is highly important to trace the origin or estimate genetic ancestry in either the respect of genetic resource protection, market surveillance or population stratification. AIMs provides a cost-effective approach compared to using whole-genome SNPs, and thus is very suitable for large-volume testing.

In the present study, we found that as few as two AIMs are sufficient to distinguish Chinese pigs from European commercial pigs, and 10 pure breeds could be accurately assigned to three corresponding groups (ECHP, SCHP and EUCP) by using as few as seven AIMs. Through data simulations, we demonstrated that the AIMs selected from unadmixed individuals can also be successfully applied to estimate ancestry proportions for admixed individuals. We further developed a panel of 129 AIMs to infer ancestry proportions in possibly admixed individuals effectively. Considering the flexibility, reliability and serviceability, Agena MassARRAY platform would be currently the best choice for genotyping for the 129 AIMs set. However, for very large-volume testing, customized low-density SNP chip or multiplex PCR-based next-generation sequencing would bemore cost-effective.

Our work provided a useful example of using a small number of AIMs for classifications and estimating ancestry proportions. Efforts can still be made to optimize the AIMs to a minimum number if necessary. For example, among the 129 AIMs, those representing the differences between EUCP and ECHP or SCHP could possibly be reduced. Or, to include more AIMs to increase the power of discrimination between ECHP and SCHP.

It is worth noting that one of the important prerequisites to obtain effective AIMs for either classification or ancestry estimation is to find good reference populations. For example, Daya et al. (2013) reported a panel of 96 AIMs could be used to infer the ancestry proportions for South African Colored (SAC) population, by using representative populations. However, these markers did not perform well in the South Asian and East Asian ancestries inference. In our study, 10 pure pig breeds from three groups (ECHP, SCHP and EUCP) are chosen as reference populations. There are several reasons why we chose these breeds. Firstly, many European commercial pigs or crossbreeding of indigenous breeds with European commercial breeds become increasingly common in China, so here major imported European commercial breeds including Duroc, Pietrain and Landrace were choosing as representative populations of EUCP. Secondly, the Chinese breeds included in this study covered two designated ancestry backgrounds. In Yang et al. study (Yang et al., 2017), China_Erhualian (CNEH), China_Jinhua (CNJH), China_Meishan (CNMS) pigs are clearly derived from one ancestry, and China_Bamaxiang (CNBX), China_Congjiangxiang (CNCJ), China_Guangdongdahuabai (CNDH) and China_Luchuan (CNLU) are clearly derived from the other. Admixture analysis showed that they are least introgressed by EUCP and can be separated from each other clearly. They together thus constitute the best reference population available so far, considering both genetic pureness and ability to reveal potential admixture in other Chinese breeds. If more pure breeds are included in the reference set in future, one could expect more accurate estimation as well as a wider range of populations where our methodcould be applicable.

## Author Contributions

YZ conceived and supervised the study. ZL analyzed the main content of the data with the assistance of LB, YQ, YP, and RY. ZL and YZ wrote the manuscript. All authors read and approved the final manuscript.

## Conflict of Interest Statement

The authors declare that the research was conducted in the absence of any commercial or financial relationships that could be construed as a potential conflict of interest.

## Footnotes

1 http://dx.doi.org/10.5061/dryad.30tk6

2 https://github.com/boxiangliu/ANTseq

3 http://www.curveexpert.net

## References

[B1] Ai H. Huang L. Ren J. (2013). Genetic diversity, linkage disequilibrium and selection signatures in chinese and western pigs revealed by genome-wide SNP markers. *PLoS One* 8:e56001. 10.1371/journal.pone.0056001 23409110 PMC3567019

[B2] Alexander D. H. Novembre J. Lange K. (2009). Fast model-based estimation of ancestry in unrelated individuals. *Genome Res.* 19 1655–1664. 10.1101/gr.094052.109 19648217 PMC2752134

[B3] Barbosa F. B. Cagnin N. F. Simioni M. Farias A. A. Torres F. R. Molck M. C. (2017). Ancestry informative marker panel to estimate population stratification using genome-wide human array. *Ann. Hum. Genet.* 81 225–233. 10.1111/ahg.12208 28895130

[B4] Bauchet M. McEvoy B. Pearson L. N. Quillen E. E. Sarkisian T. Hovhannesyan K. (2007). Measuring european population stratification with microarray genotype data. *Am. J. Hum. Genet.* 80 948–956. 10.1086/513477 17436249 PMC1852743

[B5] Berthouly-Salazar C. Thevenon S. Van T. N. Nguyen B. T. Pham L. D. Chi C. V. (2012). Uncontrolled admixture and loss of genetic diversity in a local vietnamese pig breed. *Ecol. Evol.* 2 962–975. 10.1002/ece3.229 22837841 PMC3399162

[B6] Bertolini F. Galimberti G. Schiavo G. Mastrangelo S. Di Gerlando R. Strillacci M. G. (2017). Preselection statistics and random forest classification identify population informative single nucleotide polymorphisms in cosmopolitan and autochthonous cattle breeds. *Animal* 12 12–19. 10.1017/S1751731117001355 28643617

[B7] Bianco E. Soto H. W. Vargas L. Perez-Enciso M. (2015). The chimerical genome of Isla del Coco feral pigs (Costa Rica), an isolated population since 1793 but with remarkable levels of diversity. *Mol. Ecol.* 24 2364–2378. 10.1111/mec.13182 25827466

[B8] Bosse M. Madsen O. Megens H. J. Frantz L. A. F. Paudel Y. Crooijmans R. P. (2015). Hybrid origin of european commercial pigs examined by an in-depth haplotype analysis on chromosome 1. *Front. Genet.* 5:442. 10.3389/Fgene.2014.00442 25601878 PMC4283659

[B9] Bosse M. Megens H. J. Frantz L. A. F. Madsen O. Larson G. Paudel Y. (2014). Genomic analysis reveals selection for asian genes in european pigs following human-mediated introgression. *Nat. Commun.* 5:4392. 10.1038/Ncomms5392 25025832 PMC4225517

[B10] Bouchemousse S. Liautard-Haag C. Bierne N. Viard F. (2016). Distinguishing contemporary hybridization from past introgression with postgenomic ancestry-informative SNPs in strongly differentiated Ciona species. *Mol. Ecol.* 25 5527–5542. 10.1111/mec.13854 27662427

[B11] Browning S. R. Browning B. L. (2007). Rapid and accurate haplotype phasing and missing-data inference for whole-genome association studies by use of localized haplotype clustering. *Am. J. Hum. Genet.* 81 1084–1097. 10.1086/521987 17924348 PMC2265661

[B12] Da Mota B. Tudoran R. Costan A. Varoquaux G. Brasche G. Conrod P. (2014). Machine learning patterns for neuroimaging-genetic studies in the cloud. *Front. Neuroinform.* 8:31. 10.3389/Fninf.2014.00031 24782753 PMC3986524

[B13] Dai F. W. Feng D. Y. Cao Q. Y. Ye H. Zhang C. M. Xia W. G. (2009). Developmental differences in carcass, meat quality and muscle fibre characteristics between the landrace and a Chinese native pig. *S. Afr. J. Anim. Sci.* 39 267–273.

[B14] Daya M. van der Merwe L. Galal U. Moller M. Salie M. Chimusa E. R. (2013). A panel of ancestry informative markers for the complex five-way admixed South African coloured population. *PLoS One* 8:e82224. 10.1371/journal.pone.0082224 24376522 PMC3869660

[B15] Dimauro C. Nicoloso L. Cellesi M. Macciotta N. P. P. Ciani E. Moioli B. (2015). Selection of discriminant SNP markers for breed and geographic assignment of Italian sheep. *Small Rumin. Res.* 128 27–33. 10.1016/j.smallrumres.2015.05.001

[B16] Ding L. L. Wiener H. Abebe T. Altaye M. Go R. C. P. Kercsmar C. (2011). Comparison of measures of marker informativeness for ancestry and admixture mapping. *BMC Genomics* 12:622. 10.1186/1471-2164-12-622 22185208 PMC3276602

[B17] Fang M. Hu X. Jiang T. Braunschweig M. Hu L. Du Z. (2005). The phylogeny of Chinese indigenous pig breeds inferred from microsatellite markers. *Anim. Genet.* 36 7–13. 10.1111/j.1365-2052.2004.01234.x 15670125

[B18] Frantz L. A. F. Schraiber J. G. Madsen O. Megens H. J. Bosse M. Paudel Y. (2013). Genome sequencing reveals fine scale diversification and reticulation history during speciation in Sus. *Genome Biol.* 14:R107. 10.1186/Gb-2013-14-9-R107 24070215 PMC4053821

[B19] Galanter J. M. Fernandez-Lopez J. C. Gignoux C. R. Barnholtz-Sloan J. Fernandez-Rozadilla C. Via M. (2012). Development of a panel of genome-wide ancestry informative markers to study admixture throughout the americas. *PLoS Genet.* 8:e1002554. 10.1371/journal.pgen.1002554 22412386 PMC3297575

[B20] Getachew T. Huson H. J. Wurzinger M. Burgstaller J. Gizaw S. Haile A. (2017). Identifying highly informative genetic markers for quantification of ancestry proportions in crossbred sheep populations: implications for choosing optimum levels of admixture. *BMC Genet.* 18:80. 10.1186/s12863-017-0526-2 28836937 PMC5571632

[B21] Gong H. Xiao S. Li W. Huang T. Huang X. Yan G. (2018). Unravelling the genetic loci for growth and carcass traits in Chinese Bamaxiang pigs based on a 1.4 million SNP array. *J. Anim. Breed. Genet.* 136 3–14. 10.1111/jbg.12365 30417949

[B22] Groenen M. A. M. (2016). A decade of pig genome sequencing: a window on pig domestication and evolution. *Genet. Sel. Evol.* 48:23. 10.1186/s12711-016-0204-2 27025270 PMC4812630

[B23] Hong J. H. Cho S. B. (2008). A probabilistic multi-class strategy of one-vs.-rest support vector machines for cancer classification. *Neurocomputing* 71 3275–3281. 10.1016/j.neucom.2008.04.033

[B24] Kwon T. Yoon J. Heo J. Lee W. Kim H. (2017). Tracing the breeding farm of domesticated pig using feature selection (*Sus scrofa*). *Asian Aust. J. Anim. Sci.* 30 1540–1549. 10.5713/ajas.17.0561 29073733 PMC5666188

[B25] Larson G. Dobney K. Albarella U. Fang M. Y. Matisoo-Smith E. Robins J. (2005). Worldwide phylogeography of wild boar reveals multiple centers of pig domestication. *Science* 307 1618–1621. 10.1126/science.1106927 15761152

[B26] Lawson D. J. Hellenthal G. Myers S. Falush D. (2012). Inference of population structure using dense haplotype data. *PLoS Genet.* 8:e1002453. 10.1371/journal.pgen.1002453 22291602 PMC3266881

[B27] Lee S. Epstein M. P. Duncan R. Lin X. H. (2012). Sparse principal component analysis for identifying ancestry-informative markers in genome-wide association studies. *Genet. Epidemiol.* 36 293–302. 10.1002/gepi.21621 22508067 PMC3596262

[B28] Li C. X. Pakstis A. J. Jiang L. Wei Y. L. Sun Q. F. Wu H. (2016). A panel of 74 AISNPs: improved ancestry inference within Eastern Asia. *Forensic Sci. Int. Genet.* 23 101–110. 10.1016/j.fsigen.2016.04.002 27077960

[B29] Li S.-J. Yang S.-H. Zhao S.-H. Fan B. Yu M. Wang H.-S. (2004). Genetic diversity analyses of 10 indigenous Chinese pig populations based on 20 microsatellites. *J. Anim. Sci.* 82 368–374. 10.2527/2004.822368x 14974533

[B30] Maples B. K. Gravel S. Kenny E. E. Bustamante C. D. (2013). RFMix: a discriminative modeling approach for rapid and robust local-ancestry inference. *Am. J. Hum. Genet.* 93 278–288. 10.1016/j.ajhg.2013.06.020 23910464 PMC3738819

[B31] Monzon J. Kays R. Dykhuizen D. E. (2014). Assessment of coyote-wolf-dog admixture using ancestry-informative diagnostic SNPs. *Mol. Ecol.* 23 182–197. 10.1111/mec.12570 24148003 PMC3899836

[B32] Pardo-Seco J. Martinon-Torres F. Salas A. (2014). Evaluating the accuracy of AIM panels at quantifying genome ancestry. *BMC Genomics* 15:543. 10.1186/1471-2164-15-543 24981136 PMC4101176

[B33] Patterson N. Price A. L. Reich D. (2006). Population structure and eigenanalysis. *PLoS Genet.* 2:e190. 10.1371/journal.pgen.0020190 17194218 PMC1713260

[B34] Peterson R. E. Edwards A. C. Bacanu S. A. Dick D. M. Kendler K. S. Webb B. T. (2017). The utility of empirically assigning ancestry groups in cross-population genetic studies of addiction. *Am. J. Addict.* 26 494–501. 10.1111/ajad.12586 28714599 PMC5646819

[B35] Qin P. Li Z. Jin W. Lu D. Lou H. Shen J. (2014). A panel of ancestry informative markers to estimate and correct potential effects of population stratification in Han Chinese. *Eur. J. Hum. Genet.* 22 248–253. 10.1038/ejhg.2013.111 23714748 PMC3895631

[B36] Rosenberg N. A. Li L. M. Ward R. Pritchard J. K. (2003). Informativeness of genetic markers for inference of ancestry. *Am. J. Hum. Genet.* 73 1402–1422. 10.1086/380416 14631557 PMC1180403

[B37] Santos H. C. Horimoto A. V. R. Tarazona-Santos E. Rodrigues-Soares F. Barreto M. L. Horta B. L. (2016). A minimum set of ancestry informative markers for determining admixture proportions in a mixed American population: the Brazilian set. *Eur. J. Hum. Genet.* 24 725–731. 10.1038/ejhg.2015.187 26395555 PMC4930091

[B38] Shriver M. D. Parra E. J. Dios S. Bonilla C. Norton H. Jovel C. (2003). Skin pigmentation, biogeographical ancestry and admixture mapping. *Hum. Genet.* 112 387–399. 10.1007/s00439-002-0896-y 12579416

[B39] Sun K. Ye Y. Luo T. Hou Y. (2016). Multi-InDel analysis for ancestry inference of sub-populations in china. *Sci. Rep.* 6:39797. 10.1038/srep39797 28004788 PMC5177877

[B40] Tian C. Kosoy R. Nassir R. Lee A. Villoslada P. Klareskog L. (2009). European population genetic substructure: further definition of ancestry informative markers for distinguishing among diverse european ethnic groups. *Mol. Med.* 15 371–383. 10.2119/molmed.2009.00094 19707526 PMC2730349

[B41] vonHoldt B. M. Kays R. Pollinger J. P. Wayne R. K. (2016). Admixture mapping identifies introgressed genomic regions in North American canids. *Mol. Ecol.* 25 2443–2453. 10.1111/mec.13667 27106273

[B42] Yang B. Cui L. L. Perez-Enciso M. Traspov A. Crooijmans R. P. M. A. Zinovieva N. (2017). Genome-wide SNP data unveils the globalization of domesticated pigs. *Genet. Sel. Evol.* 49:71. 10.1186/s12711-017-0345-y 28934946 PMC5609043

[B43] Zeng X. P. Chakraborty R. King J. L. Larue B. Moura-Neto R. S. Budowle B. (2016). Selection of highly informative SNP markers for population affiliation of major US populations. *Int. J. Legal Med.* 130 341–352. 10.1007/s00414-015-1297-9 26645290

[B44] Zhang C. Lin D. Wang Y. Peng D. Li H. Fei J. (2019). Widespread introgression in Chinese indigenous chicken breeds from commercial broiler. *Evol. Appl.* 12 610–621. 10.1111/eva.12742 30828377 PMC6383742

[B45] Zhang F. Zhang L. Deng H. W. (2009). A PCA-based method for ancestral informative markers selection in structured populations. *Sci. Chin. Series C Life Sci.* 52 972–976. 10.1007/s11427-009-0128-y 19911134 PMC2920624

[B46] Zhao P. Yu Y. Feng W. Du H. Yu J. Kang H. (2018). Evidence of evolutionary history and selective sweeps in the genome of Meishan pig reveals its genetic and phenotypic characterization. *Gigascience* 7. 10.1093/gigascience/giy058 29790964 PMC6007440

[B47] Zhu Y. Li W. Yang B. Zhang Z. Ai H. Ren J. (2017). Signatures of selection and interspecies introgression in the genome of chinese domestic pigs. *Genome Biol. Evol.* 9 2592–2603. 10.1093/gbe/evx186 29016799 PMC5632314

