# Supplementary material for: Selection of Optimal Ancestry Informative Markers for Classification and Ancestry Proportion Estimation in Pigs
Source: Front Genet. 2019 Mar 11;10:183. doi: 10.3389/fgene.2019.00183 (PMC6421339; doi:10.3389/fgene.2019.00183)
Supplement: Supplementary file 1 [file Table_1.docx]

**Selection of Optimal Ancestry Informative Markers for Classification and Ancestry Proportion Estimation in Pigs**

**Supplemental Information**

*Includes:*

Supplemental Figure Legends (Figures S1-S5)

Supplemental Figures (Figures S1-S5)

Supplemental Tables (Tables S1-S8)

**Figure S Legends**

**Figure S1. Cross-validation error for K = 3-15 in the ADMIXTURE analysis.**

K=11 is the most optimal value, and the corresponding minimum cross-validation error = 0.4678.

**Figure S2. MMCC curves from *F_ST_* and *I_n_* for three paired groups.**

ECHP, East China pig; SCHP, South China pig; EUCP, European Commercial pig.

**Figure S3. Venn diagram depicting candidate AIMs overlap.**

Top ranked 200 AIMs were extracted from ~60 K chip data based on the measure of pairwise *F_ST_* in each of the three paired groups: ECHP vs. EUCP, SCHP vs. EUCP, and ECHP vs. SCHP.

**Figure S4. Curve fitting for the CV of the three ancestry proportions for simulated individuals from the number of AIMs 82 through 403.**

All of **the three** ancestry proportions are well fitted against the number of AIMs from 82 through 403 with a reciprocal logarithmic function (black lines). (A)The ancestry proportion of ECHP (green filled circle). (B) The ancestry proportion of SCHP (red filled diamond). (C)The ancestry proportion of EUCP (blue filled triangle point-up).

**Figure S5. PCA in reference data set by using 129 AIMs.**

**
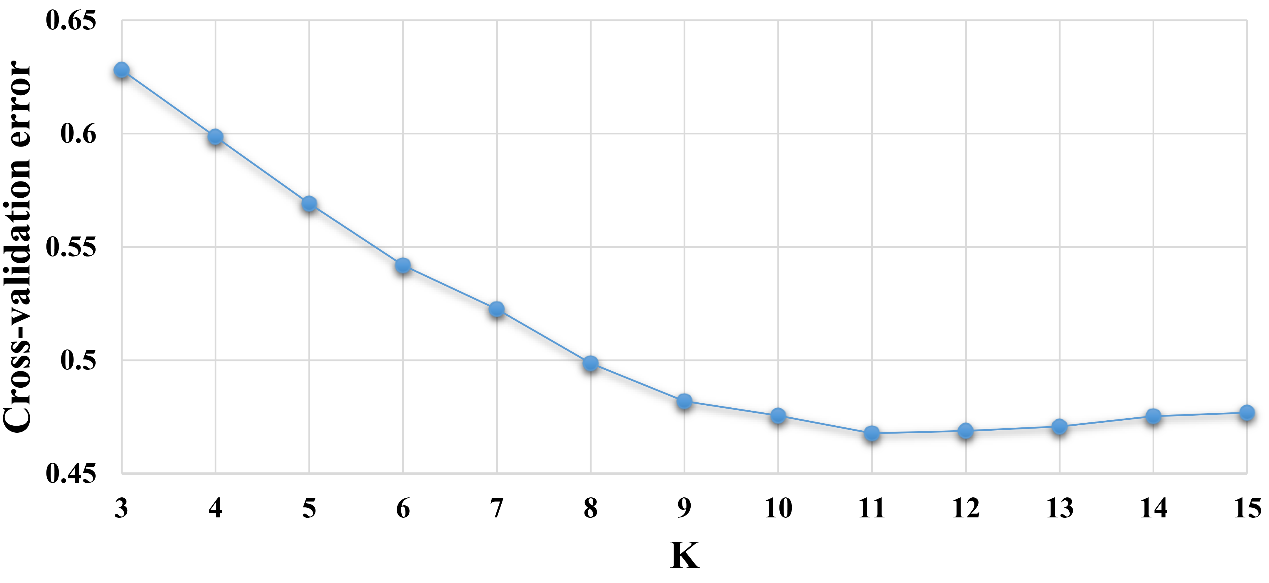
Figure S1. Cross-validation error for K = 3 through K=15 in the ADMIXTURE analysis.**


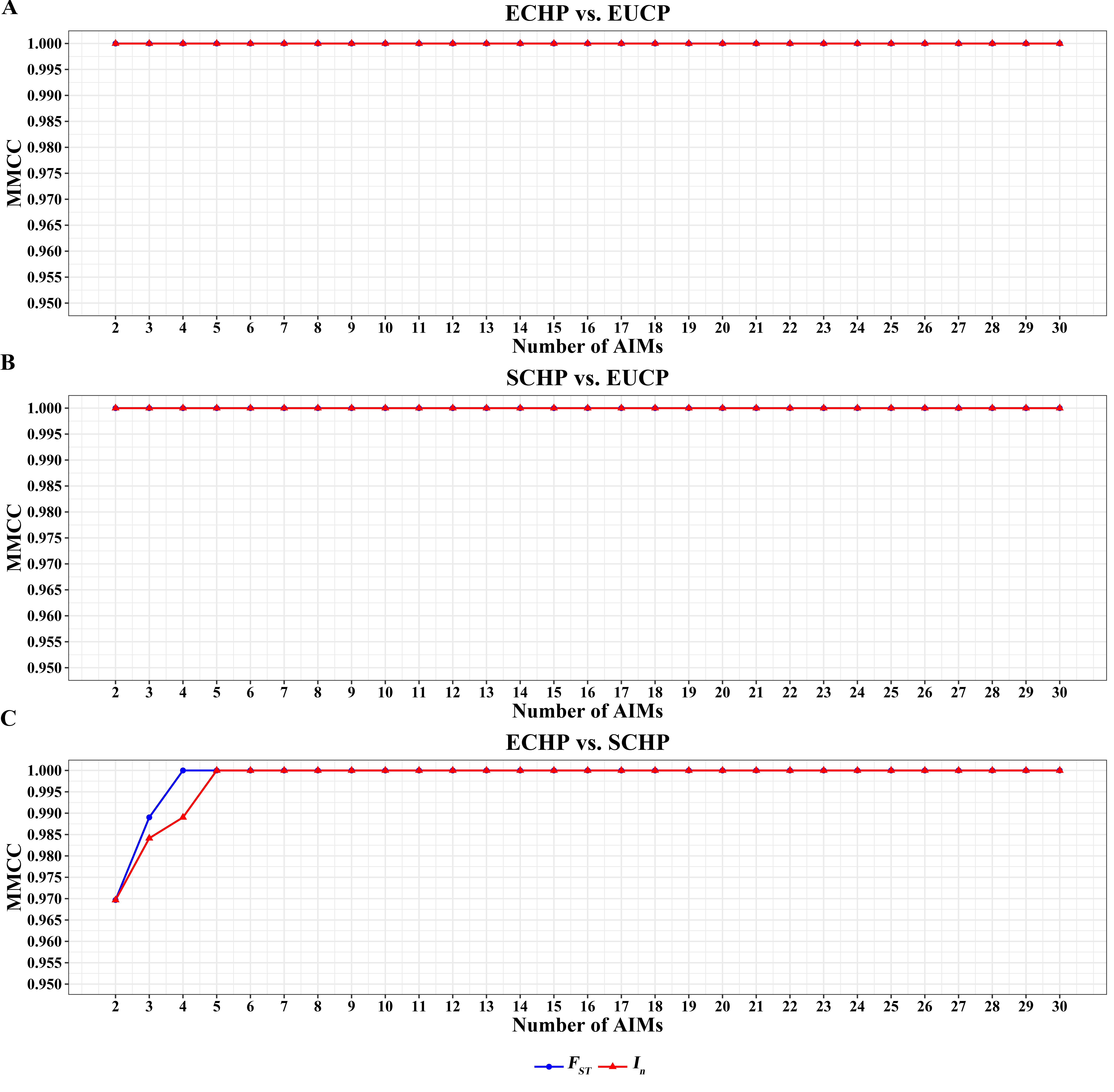


**Figure S2. MMCC curves from** ***F_ST_* and *I_n_* for three paired groups.**


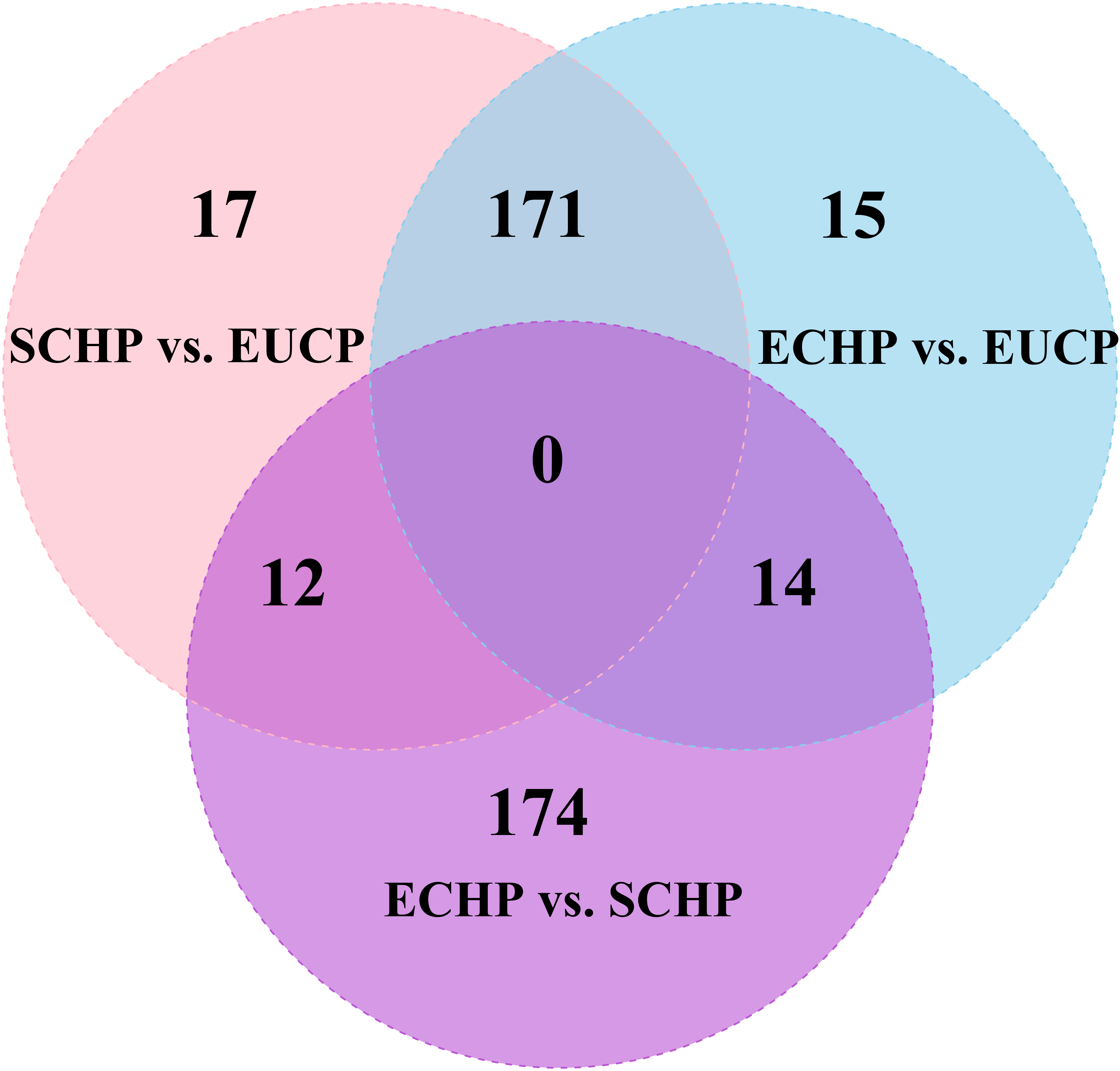


**Figure S3. Venn diagram depicting candidate AIMs overlap.**


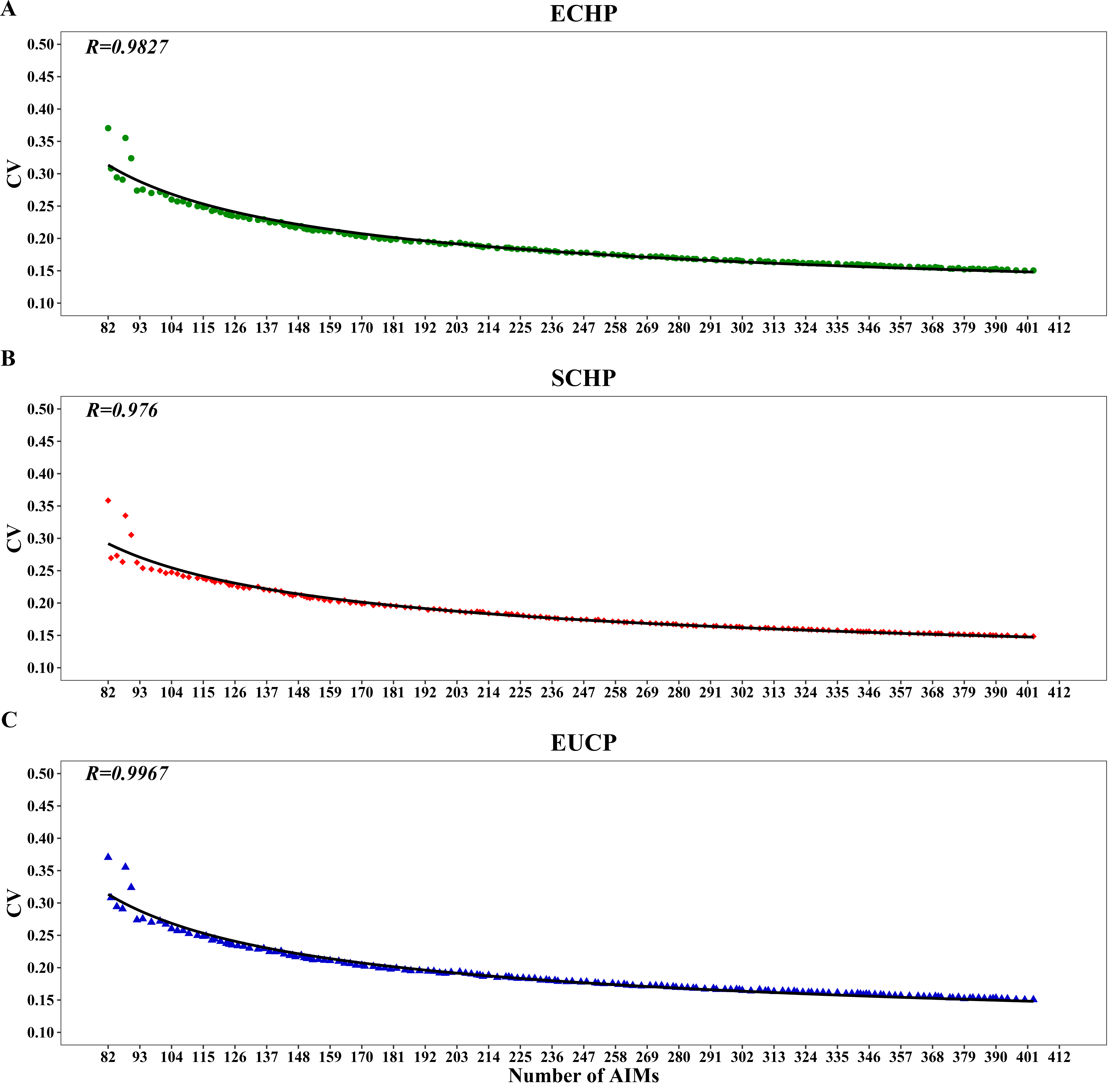


**Figure S4. Curve fitting for the CV of the three ancestry proportions for simulated individuals from the number of AIMs 82 through 403.**


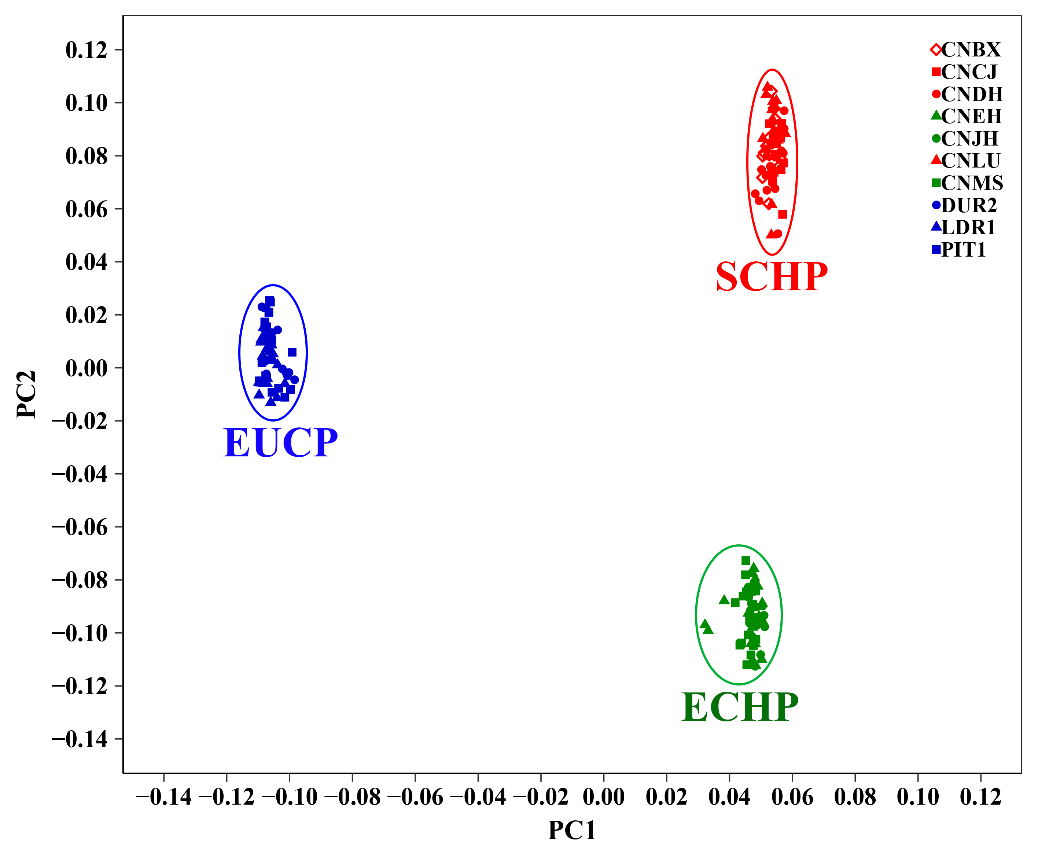


**Figure S5. PCA in reference data set by using 129 AIMs.**
